# The Impact of ACLS Training in the Management of Cardiac Arrest: A Narrative Review

**DOI:** 10.3390/epidemiologia6040061

**Published:** 2025-10-06

**Authors:** Pasquale Di Fronzo, Giovanni Gaetti, Daniel Marcassa, Valeria Gervasi, Oumaiema Dardour, Andrea Pedretti, Luca Gambolò

**Affiliations:** SIMED-Società Italiana di Medicina e Divulgazione Scientifica, 43121 Parma, Italy

**Keywords:** heart arrest, cardiac arrest, cardiopulmonary resuscitation, code blue, advanced cardiac life support, mortality

## Abstract

Background: Cardiac arrests can occur both in and out of hospital settings. Over the years, several protocols have been developed to standardize the behavior of healthcare professionals called upon to deal with these emergencies. Advanced Cardiac Life Support (ACLS) algorithms enable healthcare professionals to effectively manage cardiac arrest and achieve better patient outcomes, particularly at the time of discharge. Methods: We conducted a narrative review. Three databases (PubMed, Embase, Cochrane) were searched for relevant articles. The articles were screened and analyzed in accordance with the PRISMA guidelines. Results: A total of 1252 articles were initially identified. After screening, 11 papers were included in the review. From the selected studies, it has emerged that ACLS training had several positive effects, including an overall decrease in mortality rates. Adherence to ACLS protocols throughout an event is associated with increased Return of Spontaneous Circulation (ROSC) in the setting of In-Hospital Cardiac Arrest (IHCA). Advanced Life Support (ALS) response interval in out-of-hospital cardiac arrest was associated with decreased survival and a favorable neurological outcome. ALS response ≤ 10 min was associated with improved survival and favorable neurological outcomes. Conclusions: This review underscores the importance of adherence to ALS/ACLS guidelines in the resuscitation of patients who suffer in-hospital and out-of-hospital cardiac arrest.

## 1. Introduction

Cardiac arrest is one of the major health problems in Europe, as it affects more than 400,000 people each year, accounting for thousands of deaths. Every 90 s, an attempt at Cardiopulmonary Resuscitation (CPR) is unsuccessful because it is started too late. Even though around 70% of cardiac arrests occur in the presence of someone who could initiate CPR, bystanders initiate CPR in only 15% of cases [1]. Alarmingly, one large-scale study reported that laypersons performed CPR in just 2.9% of incidents [2].

In Italy, about 60,000 people are affected by cardiac arrest each year, in the absence of any premonitory sign or symptom. For this reason, it is crucial to train the population in early recognition and early intervention for cardiac arrests, as early intervention can reduce mortality. Among the most frequent causes of ACC are myocardial infarction, cardiac arrhythmias, drowning, electrocution, asphyxiation, trauma, and poisoning [3,4,5].

Research has shown that patients who experience cardiac arrest due to pulseless ventricular fibrillation (VF) or pulseless ventricular tachycardia (VT) have a higher survival rate compared to those with pulseless electrical activity (PEA) or asystole. In cases of PEA and asystole, defibrillation is not an appropriate treatment [5].

Chest pain, typically associated with myocardial ischemia, is commonly described with retrosternal oppressive characteristics that do not change with respiratory acts and postural changes [6,7].

Basic Life Support (BLS) and Basic Life Support and Defibrillation (BLSD) are a set of essential first aid procedures and techniques used to save a human life in the event of cardiac arrest, which can be performed by trained individuals [8].

Advanced Cardiovascular Life Support (ACLS) and Advanced Life Support (ALS) refer to structured sets of evidence-based interventions used in the management of cardiac arrest and other life-threatening cardiovascular emergencies. Advanced Cardiovascular Life Support, developed by the American Heart Association [8], is widely adopted in the United States and many other countries, while ALS is the equivalent protocol used in Europe and internationally, following the guidelines of the European Resuscitation Council [9]. Both protocols build upon Basic Life Support (BLS) by integrating advanced airway management, rhythm recognition, defibrillation, and pharmacologic therapy.

When this condition is not dealt with in the time and ways prescribed by the ACLS algorithms, the patient can rapidly progress to a cardiac arrest; in fact, the area of ischemia can generate necrotic areas on which arrhythmias such as VF can be established [10]. Following the development of a cardiac arrest, in the absence of circulation the lack of oxygen to the brain produces lesions that can quickly become irreversible as soon as 4–6 min after the absence of circulation [11].

First rescue maneuvers in CPR are external chest compressions, which should be performed as soon as possible, as they are one of the main elements that promote the survival of a person with cardiac arrest. In addition to CPR, if available, the use of a Semi-Automatic External Defibrillator (AED) allows non-healthcare personnel to deliver an electric shock when indicated by the defibrillator [12,13].

In the absence of CPR, the effectiveness of AED decreases by 7–10% for each minute of delay, whereas with CPR, the chances of survival decrease by only 3–5% after each minute since the onset of cardiac arrest. With effective cardiac massage, a cardiac output of 17% to a maximum of 25% can be ensured [14].

The achievement of Return of Spontaneous Circulation (ROSC) is an important outcome in the management of cardiac arrest, as it can greatly improve the patient’s chances of survival. Continuous monitoring and appropriate care after ROSC achievement are critical to successful treatment and optimizing the patient’s recovery [8,15].

It is crucial to highlight that drug administration during ALS should be performed by trained health care providers and should follow the specific algorithms for each cardiovascular emergency. In addition, the doses and administration indications may vary depending on the guidelines of the health care provider in which we are practicing, the site, and the patient’s specific clinical conditions [8,15,16,17,18,19,20].

Simulations should be considered an educational strategy that could be used to prepare students, as well as clinicians, who are unfamiliar with new clinical practice areas. The literature has highlighted the potential role of simulation in bridging the gap between theory and practice found in health care education, as it can ensure that healthcare professionals are adequately trained to handle this type of algorithm in an emergency [17,18,19,20].

Furthermore, simulation training has been effective in reducing professionals’ anxiety and in increasing theoretical knowledge [21]. Another important element is post-emergency debriefing. This element is necessary to understand any errors and issues that arise during an emergency to improve the training of all professionals [22].

Unfortunately, studies regarding the impact of simulation training on patient outcomes are lacking.

This review aims to determine whether ACLS/ALS training targeted to health care providers can be correlated with a reduction in mortality during intrahospital cardiac arrest.

## 2. Materials and Methods

### 2.1. Research Concept

This is a narrative review. The research concept was defined through the “PICO” framework as follows [23]: P (in-hospital and out-of-hospital cardiac arrest patients); I (ACLS training); C (No ACLS training); O (ROSC, Survival, Quality of Life). The review question was articulated as follows: Is ACLS/ALS training in healthcare personnel correlated with a reduction in mortality in cardiac arrest?

### 2.2. Inclusion and Exclusion Criteria

The following inclusion and exclusion criteria were established:

Inclusion criteria

- Patients aged 18 years and older;
- Intrahospital setting (IHCA);
- Out-of-hospital setting (OHCA);
- Cardiac arrest.

Exclusion criteria:

- Age younger than 18 years;
- Trauma patients.

### 2.3. Research Strategy

A research string was developed using the keywords Heart Arrest, Cardiac Arrest, Cardiopulmonary Resuscitation, Code Blue, Advanced Cardiac Life Support, and Mortality linked by the Boolean operators “AND” and “OR”. A detailed search strategy can be found in Appendix A. The research string was converted and used in PubMed, Embase, and Cochrane databases. The search on databases ended on 24 July 2024. All papers were uploaded to Rayyan (http://rayyan.qcri.org), a free web and mobile app used by researchers to expedite the initial screening of articles [24].

Studies were screened by title and abstract by two independent researchers (AP and OD). A third researcher (VG) participated in the discussion and resolved any conflicts. A fourth researcher (DM) read the articles selected by the group in full text and selected those relevant to the research question.

Data were extracted from a pre-established Excel database.

Extracted information included authors, year of publication, journal, country, setting of cardiac arrest (OHCA/IHCA), sample size, measured outcomes, and main results.

## 3. Results

### Main Findings

**Figure 1:**
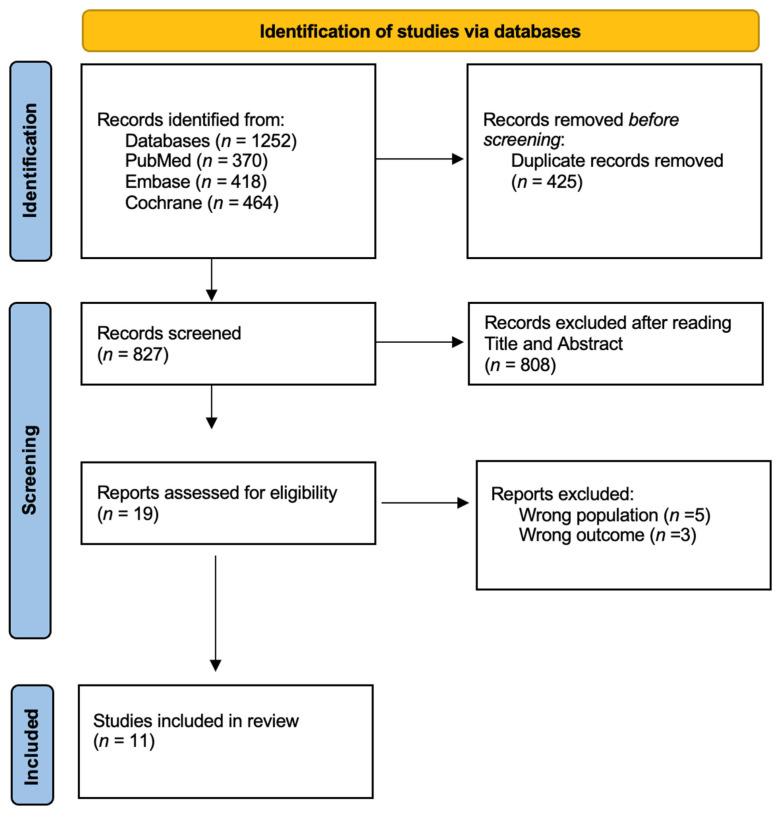
PRISMA flow diagram of studies included in the review.

A total of 1252 articles were initially identified, including 370 from PubMed, 418 from Embase, and 464 from Cochrane. After the removal of duplicates, a total of 827 articles were screened by title and abstract. After the first screening,48 articles were selected, and 779 were excluded from the study. After full-text screening, a total of 11 papers were included in the review and summarized in Table 1. The selection phase is shown in Figure 1 according to the PRISMA statement [25]. Considering the journals of publication, 6 articles (54%) were published in Resuscitation [26,27,28,29,30,31], while the rest of the articles were published in the European Journal of Cardiovascular Nursing [32], Annals of emergency medicine [33], Critical care medicine [34], Indian journal of Anaesthesia [35] and PlosOne [36].

The selected studies covered a wide geographical area, spanning North America [26,27,29,31,33,34], South America [30], Asia [35], and Europe [28,32,36]. The majority of selected studies (7, 63%) analyzed intrahospital cardiac arrest (IHCA) [29,30,31,32,33,34,35], while 4 studies focused on out-of-hospital cardiac arrest [26,27,28,36]. Overall, a total of 50.978 patients were analyzed, of which around 70% from the Kurz et al. study [26].

The studies included in this review were published between 1994 and 2020. Most of them were published in 2018 (4 studies, 36.4%) [26,27,29,35], followed by 2 studies in 2007 (18.2%) [28,30]. The remaining publications were evenly distributed across the years 1994, 1997, 2013, 2019, and 2020, each contributing one study (9.1%) [31,32,33,34,36]. In terms of geographical distribution, the majority of studies were conducted in the United States (4 studies, 36.4%) [26,31,33,34], followed by Canada with 2 studies (18.2%) [27,29]. One study (9.1%) was conducted in Italy [28], India [35], Brazil [30], and Switzerland [36]. Additionally, one study was a European multicenter study involving 12 countries [32], and was not attributed to a single nation.

Regarding study design, the majority of included studies (63.6%) were retrospective [26,28,29,30,31,33,34]. One study (9.1%) employed an observational cross-sectional design [32], and another was a secondary analysis of cohort data [27]. Two studies (18.2%) used a prospective multicenter approach [28,30]. This distribution highlights a predominance of retrospective methodologies in the literature, with relatively fewer prospective or analytically designed studies available.

Among the 11 studies included, return of spontaneous circulation (ROSC) was the most frequently reported outcome, analyzed in 9 studies (81.8%) [26,28,29,30,32,33,34,35]. Survival to hospital discharge was evaluated in 8 studies (72.7%) [26,27,28,30,33,34,35], while favorable neurological outcomes were reported in 2 studies (18.2%) [26,27]. Only one study (9.1%) assessed long-term survival at 30 days and 1 year [30]. Five studies (45.5%) examined the impact of knowledge or adherence to ACLS guidelines [29,31,32,33,34], and four studies (36.4%) specifically evaluated the effects of structured ACLS or BLS training programs on resuscitation outcomes [30,33,34,35]. ROSC and survival outcomes were consistently higher in contexts with better adherence to ACLS protocols, structured training, or timely ALS interventions.

## 4. Discussion

This narrative review examined the impact of ALS/ACLS training on clinical outcomes following in-hospital (IHCA) and out-of-hospital cardiac arrest (OHCA). The included studies consistently support the positive influence of structured resuscitation training on key clinical endpoints, particularly return of spontaneous circulation (ROSC) and survival to discharge. Nine studies (81.8%) reported data on ROSC, and eight (72.7%) assessed survival to hospital discharge. In all these cases, improved outcomes were associated with the presence of ALS/ACLS-trained personnel, increased adherence to guidelines, or the implementation of formal training programs [26,27,28,29,30,32,33,34,35].

The data suggest that ALS/ACLS training enhances both the technical and cognitive performance of healthcare providers during resuscitation. One study demonstrated a significant increase in ROSC rates (from 19.7% to 30.1%) after nurses received ACLS training [35], while another found that higher test scores on ALS knowledge assessments were directly correlated with increased ROSC among IHCA patients [32]. In a different setting, fewer deviations from ACLS algorithms during resuscitation were strongly associated with both increased ROSC and survival to discharge [29,31]. Moreover, studies assessing the timing of ALS interventions reported improved neurological outcomes and survival when ALS was initiated within 10 min of arrest [26,27]. Beyond formal training, the continuous implementation and reinforcement of evidence-based resuscitation guidelines have likewise been demonstrated to improve patient outcomes [36].

While most of the reviewed studies focused on short-term outcomes, one multicenter cohort also reported improvements in survival at 30 days and 1 year when ACLS-trained team members were present [30], suggesting that the impact of training may extend beyond the acute phase of care. However, long-term data remain limited, and future studies should further investigate sustained benefits, including neurological recovery and quality of life after discharge.

It is very interesting to note that there are no research studies in recent years on these issues; this may be due to several elements, especially the impact of the pandemic caused by COVID-19 [37]. This had a great impact on the emergency system, leading to a marked reduction [38] in training projects and training courses. Therefore, it is important to stimulate this kind of training and research projects in these years.

Furthermore, during the COVID-19 pandemic, training methods underwent significant changes, leading to new models that enhanced healthcare professionals’ autonomy. In this context, it is crucial to continue research on this topic to understand and optimize these developments [39].

The implications of these findings are substantial for healthcare systems and policymakers. Training interventions represent a relatively low-cost, scalable strategy to improve the effectiveness of resuscitation and reduce preventable mortality. As highlighted by Descatha et al. in their systematic review of workplace cardiac arrests, early and well-executed resuscitation efforts, which are made possible by trained personnel, could significantly reduce both death rates and long-term healthcare costs [11]. These observations underscore the imperative for national health authorities to implement and mandate regular ALS/ACLS certification and recertification for all clinical staff involved in emergency and acute care. In the context of life-saving interventions, such training is not optional but a core component of clinical responsibility. Repetition of key skills and algorithms through structured recertification is essential—not redundant—particularly in high-risk environments such as emergency departments, intensive care units, and prehospital emergency services. The evidence consistently demonstrates that ALS/ACLS training has a measurable and clinically significant impact on survival outcomes following cardiac arrest. Embedding standardized resuscitation training into institutional protocols and routine professional development pathways represents a high-yield, ethically grounded intervention to improve patient outcomes and ensure readiness in time-critical situations.

## 5. Conclusions

This review underscores the importance of adherence to ALS/ACLS guidelines in the resuscitation of patients who suffer in-hospital and out-of-hospital cardiac arrest. Ongoing implementation of resuscitation guidelines, in conjunction with structured ALS/ACLS training, emerges as a critical strategy to optimize outcomes after cardiac arrest. Establishing comprehensive training for healthcare professionals to emphasize the practical applications of the ACLS algorithms, increasing simulation-based training models, debriefing after events, and integrating real-time feedback following resuscitation events. The narrative review of the literature indicates that certain patient outcomes improve when care is provided by professionals who have completed specific advanced life support training. This finding carries important public health implications and should be carefully considered by all relevant stakeholders. Strengthening and expanding access to standardized training programs such as ALS and ACLS represents a critical step toward improving survival rates and reducing adverse outcomes following cardiac arrest.

This review has several limitations. As a narrative review, it does not follow the rigorous methodological framework of a systematic review, such as protocol registration, predefined inclusion criteria, or comprehensive risk of bias assessment. As such, selection bias may have influenced the choice of studies included. The heterogeneity of the available literature, spanning different healthcare systems, study designs, and outcome measures, limits the comparability of findings and the ability to draw generalized conclusions. Causality cannot be established due to the observational nature of most included studies. Finally, there is limited data from low- and middle-income countries, which restricts the global applicability of the conclusions. Further high-quality, controlled research is necessary to better understand the specific mechanisms by which ACLS training influences outcomes and to guide policy implementation across diverse settings.

## Figures and Tables

**Table 1:** Data extraction table of studies included with main information.

First Author, Year, Country	Journal	Type of the Study	Setting	Sample	Outcomes	Main Results
Kourek, 2020, European Union [32]	European Journal of Cardiovascular Nursing	Observational, cross-sectional multicenter study	IHCA	--	Correlation between ACLS guidelines knowledge and ROSC rates in the selected hospitals	Correlation between cardiopulmonary resuscitation knowledge and ROSC rates of patients with IHCA demonstrated that each additional correct answer on the advanced life support score results in a further increase in return of spontaneous circulation rates.
Kurz, 2018, USA [26]	Resuscitation	Retrospective study	OHCA	35,065 patients	Survival to hospital discharge; prehospital ROSC; 24-h survival, and favorable neurological survival defined as a modified Rankin score ≤ 3 At hospital discharge	ALS care with or without initial BLS care was independently associated with increased adjusted ROSC and survival to hospital discharge unless delivered greater than 6 min after BLS arrival (BLS + late ALS).
Grunau, 2019, Canada [27]	Resuscitation	Secondary analysis of consecutive adult OHCAs	OHCA	12,722 patients	Survival and favorable neurological outcomes (modified Rankin scale ≤ 3) at hospital discharge	ALS response interval (per minute) was associated with decreased survival and a favorable neurological outcome. ALS response ≤ 10 min was associated with improved survival and favorable neurological outcomes. Earlier ALS arrival was associated with improved survival and favorable neurological outcomes.
Larribau, 2018, Switzerland [36]	PloS One	Retrospective observational study	OHCA	795 patients	Survival to hospital discharge; ROSC	The prognosis of patient survival at the time of hospital discharge rose from 10.33% in 2009–2010 to 17.01% in 2011–2012 (*p* = 0.007). Survival rate for OHCA patients improved significantly in 2011–2012. These data suggest that it was probably the improvement in the quality of care provided during CPR and post-cardiac arrest care that contributed to the increase in survival rates at the time of hospital discharge.
Honarmand, 2018, Canada [29]	Resuscitation	Retrospective study	IHCA	160 patients	ROSC and survival to hospital discharge	There were fewer deviations during events that led to survival to hospital discharge compared to those where the patient did not survive to hospital discharge. A higher number of deviations from ACLS guidelines during resuscitation events was associated with a lower likelihood of not only ROSC, but also survival to hospital discharge.
Pareek, 2018, India [35]	Indian Journal of Anaesthesia	Retrospective study	IHCA	632 patients	ROSC and survival to discharge	During the pre-BLS/ACLS training period of the 294 Cardiac arrest patients, 58 patients (19.7%), had ROSC, while during the post-BLS/ACLS training period, 102 patients (30.1%) of the 338 patients who had cardiac arrest had ROSC (*p* = 0.003).
McEvoy, USA, 2013 [31]	Resuscitation	Retrospective study	IHCA	149 patients	ROSC	The percentage of correct steps performed was positively correlated with ROSC from an IHCA (*p* < 0.01), and the number of errors of commission and omission were both negatively correlated with ROSC from an IHCA (*p* < 0.01).
Kette, 2007, Italy [28]	Resuscitation	Prospective, multicenter study	OHCA	194 patients	Survival to hospital discharge	Compared results of two studies of the same research group (1994–2003), the rate of VF or pulseless VT as presenting rhythm reduced with a rate of return of spontaneous circulation of 69.2% and survival to hospital discharge of 41%. Hospital discharge for asystole or pulseless electrical activity remained drab (3.1% and 1.7%).
Moretti, 2007, Brazil [30]	Resuscitation	Multicenter, prospective cohort study	IHCA	156 patients	ROSC; survival to hospital discharge, survival to 30 days, and survival to 1 year	The presence of an ACLS-trained team member at an in-hospital resuscitation event increases ROSC, short-term, and long-term survival following cardiac arrest.
Camp, 1997, USA [33]	Annals of Emergency Medicine	Retrospective study	IHCA	236 patients	Survival to hospital discharge	There was a three-period study. Before, during, and after the organization of an ACLS teaching program. There were 893 total death events in the early period and 485 in the final period. After widespread ACLS training, there was a decrease in death events.
Birnbaum, 1994, USA [34]	Critical care medicine	Case–controlled, retrospective study	IHCA	869 patients	Nurses and physicians’ behaviors; mortality rate	Rates of successful attainment of the terminal behavior objectives by physicians and nurses were 84.0% and 78.8%, respectively. The mortality rates decreased from 17.4% in the period before the training to 13.1% after the ACLS course.

## Appendix A

**Table A1:** Research strategy.

PUBMED	((heart arrest [Title/Abstract]) OR (cardiac arrest[Title/Abstract]) OR (heart arrest[MeSH Terms]) OR (cardiopulmonary resuscitation[MeSH Terms]) OR (cardiopulmonary resuscitation[Title/Abstract]) OR (blue code[title/abstract])) AND ((ALS [title/abstract]) OR (Advance life support [title/abstract]) OR (ACLS [Title/Abstract]) OR (Advanced Cardiac Life Support[MeSH Terms]) OR (Advanced Cardiac Life Support[Title/Abstract])) AND ((mortality[Title/Abstract]) OR (mortality[MeSH Terms]))
EMBASE	(‘heart arrest’: ti,ab OR ‘cardiac arrest’: ti,ab OR ‘heart arrest’/exp OR ‘cardiopulmonary resuscitation’/exp OR ‘cardiopulmonary resuscitation’: ti,ab OR ‘blue code’: ti,ab) AND (‘als’: ti,ab OR ‘advance life support’: ti,ab OR ‘acls’: ti,ab OR ‘advanced cardiac life support’/exp OR ‘advanced cardiac life support’: ti,ab) AND (‘mortality’: ti,ab OR ‘mortality’/exp)
COCHRANE	1-MeSH descriptor: [Heart Arrest] explode all trees 2882 2-‘heart arrest’: ti,ab OR ‘cardiac arrest’: ti,ab OR ‘cardiopulmonary resuscitation’: ti,ab OR ‘blue code’: ti,ab 6450 3-‘ALS’: ti,ab OR ‘Advance life support’: ti,ab OR ‘ACLS’: ti,ab OR ‘Advanced Cardiac Life Support’: ti,ab OR ‘ACLS’: ti,ab 3550 4-MeSH descriptor: [Mortality] explode all trees 21876 5-‘mortality’: ti,ab OR ‘death’: ti,ab OR ‘rosc’: ti,ab 135505 (#1 OR #2) AND (#3) AND (#4 OR #5) 143
